# Adenine oxidation by pyrite-generated hydroxyl radicals

**DOI:** 10.1186/1467-4866-11-2

**Published:** 2010-04-26

**Authors:** Corey A Cohn, Shawn C Fisher, Bruce J Brownawell, Martin AA Schoonen

**Affiliations:** 1 Center for Environmental Molecular Science, Department of Geosciences, Stony Brook University, Stony Brook, N.Y. 11794-2100 USA; 2 Marine Sciences Research Center, Stony Brook University, Stony Brook, N.Y 11794-5000 USA; 3 Office of Science, U.S. Department of Energy, Washington D.C. 20585 USA

## Abstract

Cellular exposure to particulate matter with concomitant formation of reactive oxygen species (ROS) and oxidization of biomolecules may lead to negative health outcomes. Evaluating the particle-induced formation of ROS and the oxidation products from reaction of ROS with biomolecules is useful for gaining a mechanistic understanding of particle-induced oxidative stress. Aqueous suspensions of pyrite particles have been shown to form hydroxyl radicals and degrade nucleic acids. Reactions between pyrite-induced hydroxyl radicals and nucleic acid bases, however, remain to be determined. Here, we compared the oxidation of adenine by Fenton-generated (i.e., ferrous iron and hydrogen peroxide) hydroxyl radicals to adenine oxidation by hydroxyl radicals generated in pyrite aqueous suspensions. Results show that adenine oxidizes in the presence of pyrite (without the addition of hydrogen peroxide) and that the rate of oxidation is dependent on the pyrite loading. Adenine oxidation was prevented by addition of either catalase or ethanol to the pyrite/adenine suspensions, which implies that hydrogen peroxide and hydroxyl radicals are causing the adenine oxidation. The adenine oxidation products, 8-oxoadenine and 2-hydroxyadenine, were the same whether hydroxyl radicals were generated by Fenton or pyrite-initiated reactions. Although nucleic acid bases are unlikely to be directly exposed to pyrite particles, the formation of ROS in the vicinity of cells may lead to oxidative stress.

## Background

Pyrite (FeS_2_), the most common metal sulfide mineral associated with coal and metal mine waste, has been shown to generate hydrogen peroxide (H_2_O_2_) [1,2] and hydroxyl radicals (^•^OH) [3,4] when placed in water. In the presence of dissolved molecular oxygen, ferrous iron associated with pyrite can form superoxide anion (O_2_^•^)^- ^(eq. 1), which reacts with ferrous iron to form H_2_O_2 _(eq. 2) and eventually ^•^OH (Fenton reaction, eq. 3).(1)

The formation of reactive oxygen species (ROS) such as H_2_O_2 _and ^•^OH is significant because of their reactivity; ^•^OH will typically react with nearly all molecules in aqueous solution at diffusion-limited rates [5]. Their extreme reactivity has been implicated in causing or contributing to disease and aging in humans [6-10]. Particles other than pyrite such as asbestos [11] and quartz [10] have also been shown to induce the formation of ^•^OH in lung cells that have been exposed to the particles. The particulate-induced formation of ^•^OH has been linked with oxidative stress [12,13] and genotoxicity [14,13]. Hence, ^•^OH formation *in vitro *and *in vivo *has been used as an indicator for mineral-induced toxicity potential [14,13,12,16,6].

The extremely short half-life of ^•^OH hinders detection and quantification of ^•^OH concentrations directly [5]. Instead, detection requires the reaction of ^•^OH with a target molecule. Upon reaction, characteristics of the target molecule such as light absorption [2], fluorescence [17-19], or electron spin resonance [20-23] may change. The detection of these changes is then used to determine the presence and concentration of ^•^OH and other ROS. In the presence of cells or in tissue, the products of particle-induced radical oxidation include DNA strand-breaks [24,14], RNA degradation [4], and nucleobase oxidation [25-27]. Nucleic acids react with ^•^OH by hydrogen abstraction at the sugar or addition to the bases, both resulting in radical moieties and de-polymerization [28,29,24]. Oxidized base reaction products are typically detected using chromatography and mass spectroscopy [30-33]. Reaction of ^•^OH with the purine bases guanine or adenine leads to common persistent products containing an additional single oxygen in the molecule (M+16). Examples of oxidation products generated by reaction of purine bases with ^•^OH include 8-hydroxyguanine and 8-oxoadenine, in equilibrium with its less stable tautomer 8-hydroxyadenine (see [28,34,32-37] for reviews). The reported M+16 products from reaction of adenine with ROS include 8-oxoadenine, 2-hydroxyadenine (isoguanine), and 6-N-hydroxyaminopurine (HAP) [38,39].

The bio-available iron that is associated with pyrite in coal samples has been linked to the development of coal workers pneumoconiosis (CWP) in coal miners [40,41]. Similarly, coal samples that contain pyrite have been shown to cause nucleic acid strand-breaks with an increasing degree of strand-breaks with greater pyrite content in the coal samples [4]. While nucleic acid strand-breaks can occur in the presence of pyrite, the fate of the bases in the presence of pyrite-generated ^•^OH has not been evaluated. The objective of this study was threefold: a) determine the effect of ^•^OH concentration on the stability of the nucleobase adenine; b) determine *if pyrite-generated ^•^OH degrade adenine*; and c) evaluate the adenine degradation products from reaction with pyrite.

In order to evaluate ^•^OH-induced degradation of adenine, several experiments were performed exposing adenine solutions to various reactants and pyrite suspensions. The aqueous reactants included Fenton-generated ^•^OH, the separate Fenton reagents [i.e., H_2_O_2 _and Fe(II)], and Fenton reagents with addition of catalase or ethanol. Catalase is an enzyme that reacts with H_2_O_2 _to form H_2_O and O_2_. When a high concentration of ethanol is added to a solution containing lower concentrations of adenine and Fenton reagents, ethanol will compete in scavenging ^•^OH. Hence, addition of ethanol is expected to stabilize adenine in pyrite suspensions. Batch experiments were also performed by exposing adenine solutions to pyrite particles and with the addition of ethanol or catalase. The concentrations of adenine remaining after incubation with aqueous reactants or pyrite were determined using UV-Vis spectrophotometry and the reaction products were analyzed using high-pressure liquid chromatography time-of-flight mass spectroscopy (LCTOF-MS).

## Results

**Figure 1:**
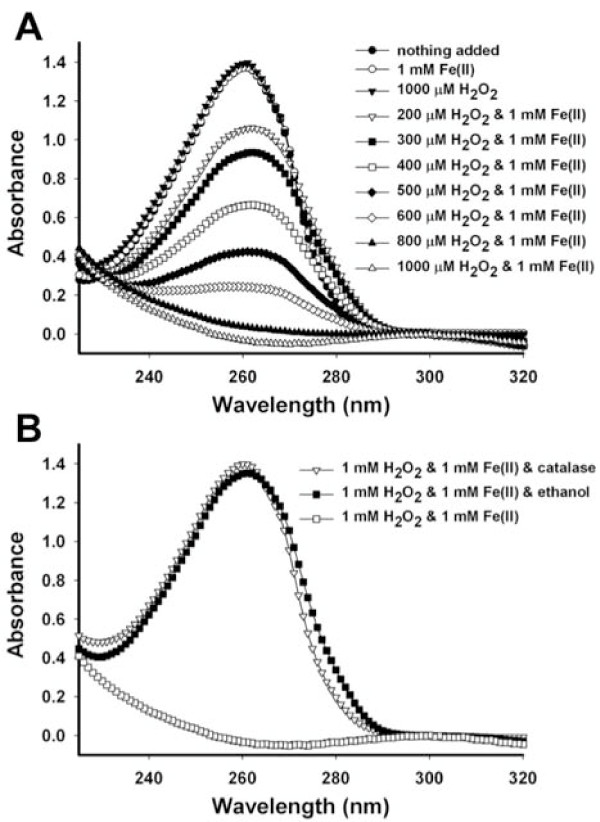
**(A) Adenine degradation by Fenton-generated hydroxyl radicals**. 100 μM adenine was mixed with either Fe(II), H_2_O_2 _or a combination of the two, which generates ^•^OH. The solutions were incubated for 24 hrs followed by wavelength scans. Loss of absorbance at 260 nm is indicative of degradation of the adenine molecule. (B) 64 kUnits catalase or 50% ethanol were added to adenine solutions with Fenton reagents. The 1 mM H_2_O_2 _& 1 mM Fe(II) plot from graph A is included for comparison. Note that the order in the legend follows the same order of curves in the graphs from top to bottom so in (A), the top three curves overlap (i.e., "nothing added", "1 mM Fe(II)", and "1000 μM H_2_O_2_").

To determine the susceptibility of adenine to ^•^OH-induced degradation, several experiments were conducted. Adenine solutions were placed in several centrifuge tubes. In some of the tubes, Fenton reagents [H_2_O_2 _& Fe(II)] were added. In other tubes, just H_2_O_2 _or Fe(II) were added. After 24 hrs of incubation wavelength scans of the solutions were recorded (Figure 1). These results show that the adenine solution, adenine & H_2_O_2_, and adenine & Fe(II) are all stable over the course of the experiment. However, when both Fenton reagents are combined, they react to form ^•^OH and the absorbance associated with adenine at 260 nm decreases. This is indicative of loss of a chromophoric property, which is caused by alteration of the bonds within adenine. Increasing the concentration of H_2_O_2 _results in greater loss of absorbance at 260 nm. Since both Fe(II) and H_2_O_2 _will absorb UV light, their absorbance curves have a higher baseline compared to a solution with only adenine. To simplify the interpretation of the data, the background absorbance from the curves in Figure 1 are all corrected so that their absorbances at 300 nm are all zero. The addition of catalase or ethanol stabilizes the adenine indicating the putative mechanistic roles of H_2_O_2 _and ^•^OH in degrading the adenine.

**Figure 2:**
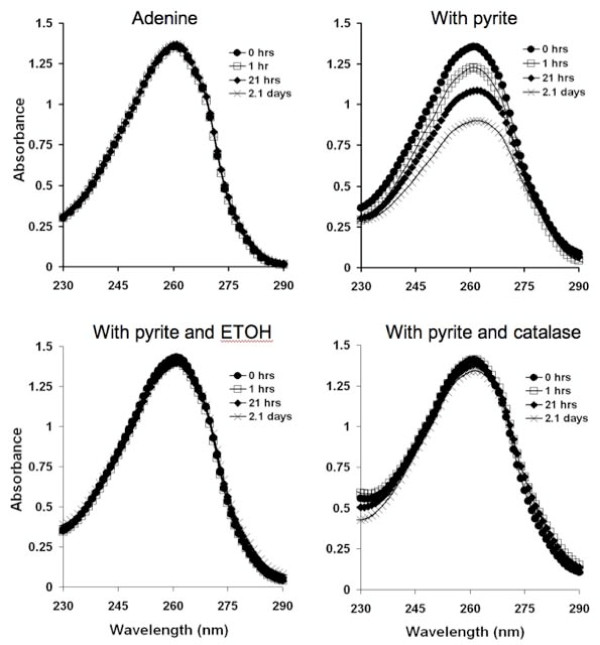
**Adenine exposed to pyrite**. 100 μM adenine was incubated and agitated with 10 g/L pyrite and samples were periodically withdrawn and filtered to remove the pyrite particles before wavelength scans were recorded. In separate vials, 50% EtOH (ethanol) and 64 kUnits catalase were added to pyrite/adenine suspensions.

**Figure 3:**
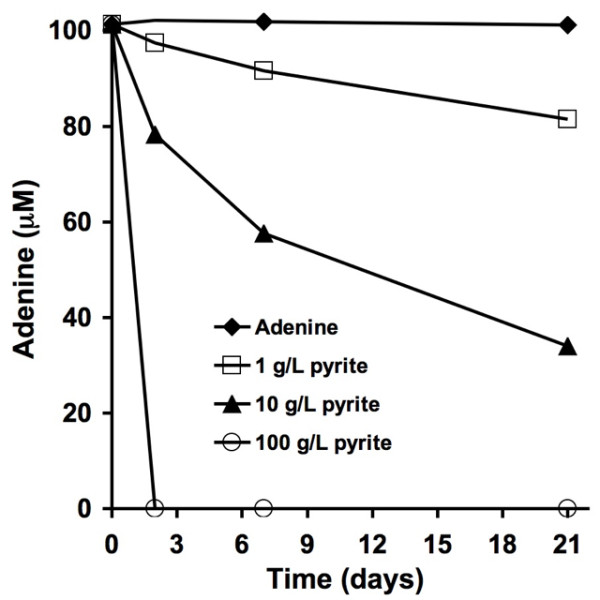
**Adenine exposed to varying pyrite particle loadings (without the addition of hydrogen peroxide)**. Adenine concentrations were determined by measuring absorbance at 260 nm from filtered samples.

Experiments were also performed to determine the stability of adenine in the presence of pyrite; specifically, the role of pyrite-generated ^•^OH in adenine degradation. When pyrite particles were added to adenine solutions the adenine/pyrite suspension show loss of absorbance centered at 260 nm (Figure 2). A decrease in absorbance may be due to either adsorption of adenine to the pyrite surface or pyrite-generated ^•^OH. In order to resolve whether ^•^OH was involved in the loss of adenine, ethanol or catalase were added separately to adenine/pyrite suspensions. The addition of either catalase (reacts with H_2_O_2 _to form H_2_O and O_2_) or ethanol (^•^OH scavenger) to adenine/pyrite suspensions resulted in a stabilization of the adenine. This supports the putative role of H_2_O_2 _and ^•^OH in the reaction with adenine. When the loading of pyrite particles in the presence of adenine is increased, the adenine degradation rate also increases (Figure 3). Although a decrease in light absorbance is indicative of a molecular structural change, spectrophotometry does not provide specific information on the adenine degradation products.

**Figure 4:**
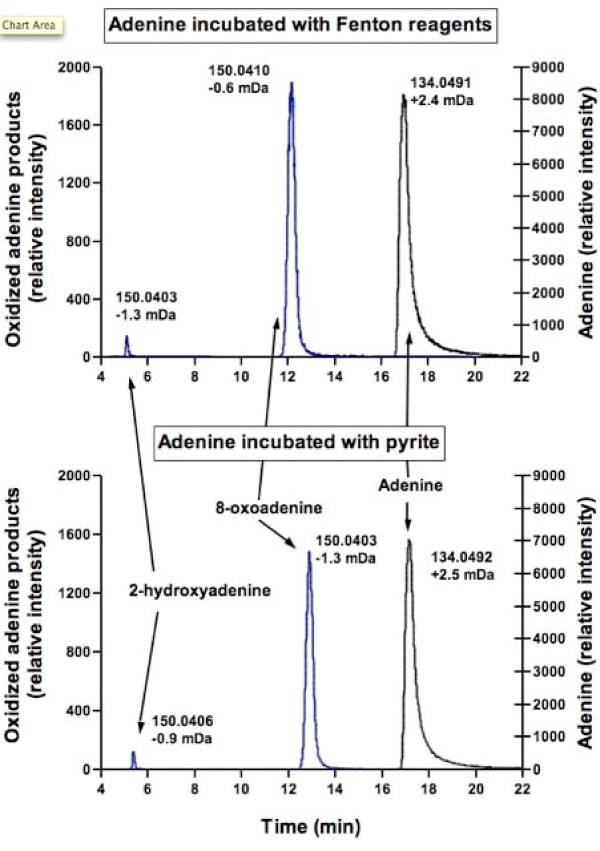
**Selected ion chromatographs in negative-ion electrospray for reactions of adenine with Fenton reagents and in presence of pyrite**. Two products result from the oxidation of adenine, 2-hydroxyadenine and 8-oxoadenine. Also indicated are the calculated accurate masses of each analyte and the difference when compared with the measured mass of the depronated parent ions.

HPLC-TOF-MS analysis was conducted on samples to characterize persistent reaction products in both pyrite and Fenton reaction treatments. Initial examination of mass chromatograms only indicated a single peak that was 16 mass units greater than the adenine parent ion, consistent with known products of adenine oxidation by ROS species. A peak with the same mass was apparent in both positive ionization mode (M+H) and negative ionization mode (M-H) and in reactions conducted both with pyrite and with Fenton reagents. No significant peaks could be detected at higher m/z that would suggest oxidative coupling or polymerization of adenine. Figure 4 illustrates the reconstructed ion chromatograms corresponding to adenine (134.0467) and adenine + oxygen (150.0416). By narrowing the mass filter (80 mDa), two M+16 peaks were apparent, both in positive and negative ionization modes. As indicated in Figure 4, the retention times of the peaks correspond to standards of 8-oxoadenine and 2-hydroxyadenine (isoguanine). Further confirmation of these peaks was provided by the accurate mass estimates provide by TOF-MS (Figure 4). Similar relative abundances of the products were observed in both pyrite and Fenton treatments. The greater accumulation of 8-oxoadenine compared to 2-hydroxyadenine (isoguanine) has also been observed in studies of adenine and adenosine reactions with hydroxyl radical [42]. 8-hydroxyadenine but not 2-hydroxyadenine was found in sonicated DNA solutions [43]. There was no observable peak in reaction mixtures corresponding to the retention time of a 6-N-hydroxyaminopurine standard (also adenine + oxygen), which has been observed as peroxyl radical oxidation product of adenine [39].

## Discussion

The aim of this study was to determine the effect of pyrite loading on the stability of adenine and to evaluate the adenine degradation products. The findings presented here show that adenine will react with both Fenton and pyrite-generated ^•^OH by addition reactions to form 8-oxoadenine and, to a lesser extent, 2-hydroxyadenine. This is consistent with previous studies where ^•^OH adds to one of the carbons in adenine followed by oxidation [28,34]. In the experiments where adenine was exposed to Fenton and pyrite-generated ^•^OH, an increase in either the Fenton reagent, H_2_O_2 _or pyrite particle loadings (i.e., greater pyrite surface area) led to higher rates of adenine degradation. The addition of either catalase or ethanol stabilized the adenine suggesting the role of H_2_O_2 _and ^•^OH, respectively. In the Fenton reaction (eq. 3), H_2_O_2 _is added to a solution containing Fe(II) resulting in the formation of ^•^OH. By adding catalase or ethanol, these species limit the availability of H_2_O_2 _and ^•^OH to react with adenine. When pyrite particles are added to a solution, we hypothesize that pyrite first forms H_2_O_2 _through a reaction involving dissolved molecular oxygen and ferrous iron (eqs 1 and 2) either with Fe(II) located at the pyrite surface or dissolved into solution. Additional Fe(II) then reacts with the H_2_O_2 _to form ^•^OH through the Fenton reaction [4]. In the experiments where catalase is added to adenine solutions with either aqueous Fe(II) or pyrite suspensions, the concentration of adenine does not change over time suggesting that the kinetics of catalase-induced removal of H_2_O_2 _is faster than the reaction whereby ^•^OH is generated via the Fenton reaction (eq. 3). Although not explored in this manuscript, it may be interesting to evaluate the independent roles of sulfur and iron oxidation in the generation of ROS and to investigate changes at mineral surface pre- and post-oxidation to evaluate the role of the mineral surface versus aqueous Fe(II) in the formation of ROS.

In addition to ^•^OH, superoxide [(O_2_^•^)^-^] also forms in the Fe(II) oxidation reactions (eqs 1 to 3) that lead to the formation of ^•^OH. While (O_2_^•^)^- ^may also oxidize adenine, the role of (O_2_^•^)^- ^compared to ^•^OH in reaction with adenine from addition of pyrite has not been supported by other experiments performed by the authors. In an experiment where we evaluated the concentration of ^•^OH in pyrite suspensions, the addition of superoxide dismutase did not have a substantial effect on the concentration of ^•^OH while the addition of catalase did result in less ^•^OH detection (data not shown).

Pyrite is the most common sulfide mineral and is present in mining waste and marine sediment. The inhalation of particles that are capable of generating ^•^OH have been linked to biomolecular oxidation [12,13] and genotoxicity [14,13]. The formation of ^•^OH by pyrite and its reaction with adenine as shown here may be relevant when pyrite particles are inhaled. For example, many sulfur-rich coals contain iron disulfide (FeS_2_) in the form of pyrite [44,45] and there is a correlation between the pyritic sulfur content in coal samples and coal workers' pneumoconiosis prevalence [46]. While this study shows that adenine is oxidized in the presence of pyrite, there are several limitations inherent in this study when extrapolating the data to other systems. This study was limited to only one of the bases, was executed with dissolved adenine instead of intact nucleic acids, and the experiments were performed in aqueous systems instead of in the presence of cells or tissues. Further experiments are necessary to determine the effect of pyrite-generated ^•^OH on the bases *in vivo*.

### Experimental Methods

#### Pyrite sample preparation

Natural pyrite (Huanzala, Peru) obtained from Wards Natural Science (Rochester, NY) was crushed in an agate mill. After crushing, the pyrite was sieved so that the collected particles were <90 μm but did not traverse the sieve with 10 μm openings. The particles were then washed with hydrochloric acid to remove surface oxides using a protocol described in earlier work [3]. A surface area of roughly 1.25 m^2^/g was determined using five-point N_2 _adsorption BET. The pyrite particles were stored in a vacuum desiccator until used in experiments.

### Adenine degradation experiments

The degradation of adenine in the presence of variable amounts of pyrite (1-100 g/L) was compared to adenine degradation in the presence of Fenton generated ^•^OH. All experiments were performed in either polypropylene opaque 2-mL or 15-mL centrifuge tubes at room temperature (23 ± 2°C). Solutions of adenine were produced by dissolving adenine powder (Sigma, 99% pure) in water (Easy Pure 18.3 MΩ-cm, UV-irradiated, and ultra-filtered) followed by filtration (Millipore PVDF 0.2 μm) and quantification (260 nm, ε = 13300 [47]). An aqueous suspension of catalase (64 kUnits final concentration) was purchased (Sigma, C100 from bovine liver), diluted in water and added to some of the tubes. Ethanol was also added to some of the tubes so that the ethanol concentration in each tube was 50% by volume. The experiments were initiated with the addition of all reagents in the tubes. The tubes were set on an end-over-end rotator. Samples taken after 24 hour incubations were taken from 2-mL tubes and samples taken as a function of time were taken from 15-mL tubes. Samples with pyrite suspensions were filtered (Millipore 13 mm 0.45 μm PVDF) before quantification of remaining adenine by UV absorption at 260 nm.

### Mass spectroscopy of the degraded adenine

Selected samples were further analyzed for identification of adenine oxidation products by HPLC-MS, utilizing electrospray ionization and a time-of-flight (TOF) mass detector (MicroMass LCT™). Separation of adenine and its oxidation products were achieved using a Waters Alliance 2695 HPLC on a Phenomonex reverse-phase C18 column (250 mm × 3.0 mm). LC-MS was conducted both in positive and negative ionization modes. Capillary and cone voltages were 2700 V and 25 V for positive ionization mode and 2200 V and 30 V in negative ion mode. The chromatography for negative ion analysis involved a solvent gradient of acetonitrile and water starting at 2% acetonitrile and increasing to 12% at 0.5% per minute for 20 minutes. For positive ion mode, the initial mobile phase composition was 5% methanol and 95% 25 μM ammonium formate solution, which after 3 minutes the percentage methanol was increased at 0.5% per minute for 30 minutes. Full spectra were continuously measured for ions between 100-800 m/z. Estimates of the accurate mass of adenine and product peaks were based on post column flow injection of the mass calibration standard leucine enkephalin and calculations performed using Masslynx™ v3.5 software (Reddy and Brownawell, 2005).

## Competing interests

The authors declare that they have no competing interests.

## Authors' contributions

CAC carried out all experiments and drafted the manuscript. SCF carried out the mass spectroscopy analyses of the degraded adenine and helped draft the manuscript sections on mass spectroscopy. BJB supervised the mass spectroscopy analyses and helped draft the manuscript sections on mass spectroscopy. MAAS participated in the design of the study, funded the study, and helped draft the manuscript. All authors read and approved the final manuscript.
